# Cost-Effective Production and Optimization of Alkaline Xylanase by Indigenous *Bacillus mojavensis AG137* Fermented on Agricultural Waste

**DOI:** 10.4061/2011/593624

**Published:** 2011-08-29

**Authors:** Abbas Akhavan Sepahy, Shokoofeh Ghazi, Maryam Akhavan Sepahy

**Affiliations:** ^1^Department of Microbiology, Faculty of Basic Sciences, Islamic Azad University, 16 South Makran St., Heravi Sq., Pasdaran, Tehran 19585, Iran; ^2^Food and Drug Control Laboratory Research Centre (FDCLRC), MOH, Tehran, Iran

## Abstract

A xylanase producer *Bacillus mojavensis* strain, called AG137, isolated from cotton farm (Kashan-Iran). The optimal xylanase activity reached at 55°C & pH 9.0. Enzyme yield was studied using a medium with different agricultural wastes as inducers. Xylanase production of about 249.308 IU/mL was achieved at pH 8 and 37°C, within 48 h submerged fermentation in enzyme production medium supplemented with 2% (w/v) oat bran as an optimum carbon source. A mixture of 1% (w/v) yeast extract and 1% (w/v) tryptone as optimum nitrogen sources, agitation speed 200 rpm, and inoculum size 2% (v/v) were the optimums for maximum production. Accordingly, xylanase yield from 194.68 IU/mL under non-optimized fermentation condition enhanced to 302.466 IU/mL in optimized condition. Screened xylanase is thermostable, presenting 70% stability at 60°C during 30 min. Further enzyme incubation in higher temperature caused a decrease in the residual enzyme activity, yet it retained 68%–50% of its activity after 1 hour from 45°C to 55°C. Besides, it is stable in pH 9 and 10, maintaining over 70% of its activity for 2 h. The enzyme also could preserve 71% and 63% of its initial activity after 3 hours of pre-incubation in the same alkaline condition. Produced xylanase therefore was introduced as an alkaline-active and stable one, displaying suitable thermostability feature, confirmed by HPLC analysis. Hence, all xylanase properties highlight its promising uses in industrial scale.

## 1. Introduction

Xylan is a major component of hemicellulose. It is a heteropolymer composed of *β*-1-4-linked D-xylose backbone and branches of arabinose, glucuronic acid, mannose, or acetyl residues [1]. Endo-*β*-1,4-xylanase is crucial for xylan depolymerisation. This ability renders xylanase a valuable tool in various biotechnological applications ranging from the food industry to textile industry [2–4]. A variety of microorganisms including* Bacillus *strains [5–7], filamentous fungi [8, 9], and actinomycetes [10] have been reported to produce xylanolytic enzymes. In the most reported cases of xylanase production from fungi and actinomycetes, the enzyme applications have been declined in most industrial processes, especially in high temperatures and alkaline pH values, due to the poor or zero xylanase activity [11, 12]. Various fungi and actinomycetes producers also have been reported to produce very low level of xylanase:* Aspergillus niger ANL* 301, with production amount of 6.47 IU/mL [11], 3.89 IU/mL xylanase unit by *Penicillium oxalicum* [13], or *Penicillium janthinellum* with 55.3 IU/mL unit of xylanase yield [14]. Xylanase production capacity therefore is needed to be amplified from bacterial sources, for bacterial xylanases almost display high optimum pH and temperature of enzyme activity and stability [3, 15, 16]. 

Consequently, investigation on novel sources of bacterial xylanase producers' strains, which display high optimal xylanase activity and stability in more drastic conditions, is still in progress. Moreover, wide-scale industrial applications of xylanase require their cost-effective production to make the process economically viable [8]. This can be achieved by using cheaply available agroindustrial residues such as wheat bran, oat bran, rice straw, or others [17]. Annually, large quantities of lignocellulosic wastes are generated through industrial processes [2]. So this can be utilized for economic production of xylanase by microorganism through fermentation processes [4, 11]. This paper reports optimization of various nutritional parameters of production medium and characterization of an alkaline xylanase from a newly indigenous strain of *Bacillus mojavensis* isolated from xylan-enriched agricultural soils owing to the fact that optimization of medium composition has to be carried out in order to maintain a balance among various medium components. In the present investigation, high-level production of alkaline-active and stable xylanase has been reported, using agroresiduals in SmF. To confirm xylanase activity, the produced xylanase was potentially applied in the selective hydrolysis of the hemicellulose component of pure oat-spelt xylan through a HPLC analysis. 

## 2. Materials and Methods

### 2.1. Materials

Oat-spelt xylan (95590) was purchased from Fluka (Darmstadt-Germany). Congo Red dye and D-xylose were purchased from Merck. All other media components and chemicals used were obtained from Sigma-Aldrich (Darmstadt-Germany). Agricultural byproducts were obtained locally from domestic market. Pretreatment of cellulosic materials was carried out by the method of Kapoor et al. [17] and Okafor et al. [11].

### 2.2. Isolation and Preliminary Screening of Xylanase-Producing Bacilli

*Bacillus *strains with xylanolytic activities were isolated by enrichment culture technique from soil of agricultural farms at Kashan, Iran. Xylan agar medium (pH 7), having (g/L): Oat-spelt xylan, 10; peptone, 5; yeast extract, 5; MgSO_4_·7H_2_O, 0.2; K_2_HPO_4_, 1; Agar, 15, was used for preliminary screening of xylanase-producing isolates. Plates then were incubated at 37°C for 48 h. Xylanase-producing bacterial colonies were selected after flooding the plates with Congo Red solution (0.1%-15 min) followed by repeated washing with (1 M) Nacl solution.

### 2.3. Secondary Screening Procedure

Xylanase-producing strains from preliminary screening were selected for enzyme plate clearing assay to detect the best extracellular xylanase producers. The harvested culture broth of each isolate, grown in xylan broth medium, described previously (pH 7), was centrifuged in a microfuge (8500 × g) for 10 min. The cell-free culture supernatants were used as the crude xylanase sources for screening purposes. Crude enzyme samples (50 *μ*L) were placed in to solidified medium containing oat-spelt xylan (3% w/v) in phosphate buffer (0.5% M, pH 8) and agar (15% w/v). A sterile 6 mm borer was used to make wells in solidified plates, and plates were incubated at 40°C for 24 h [16, 18].

Controls of heat-killed (150°C/30 min) culture supernatants also were included. Results were recorded as xylanase positive or negative and the size of the clearing zones measured for xylanase-positive strains.

### 2.4. Enzyme Production Using Submerged Fermentation

The enzyme production was studied in the Erlenmeyer flasks (250 mL), containing 50 mL of basic enzyme production medium, having (g/L): yeast extract, 5; peptone, 5; MgSO_4_·7H_2_O, 0.2; K_2_HPO_4_, 1, supplemented with wheat bran (2% w/v) in this step. Liquid enzyme production media were autoclaved, and pH was adjusted to 8.0 using sterilized Na_2_CO_3_ (10%) solution. Bacterial inocula precultures were primed in the Luria-Bertani broth medium (pH 7), having (g/L): Ttryptone, 10; yeast extract, 5; Nacl, 5. Flasks were inoculated with 2% (v/v) 18 h grown inoculum broth and incubated at 37°C under shaking condition (180 rpm) in a shaker incubator (Heidolph UNIMAX *1010*). 1 mL culture samples containing xylanase enzyme were removed after 24 h of SmF. The enzyme was harvested by centrifuging (8500 × g) for 10 min. The supernatant was treated as crude enzyme and was assayed for xylanase activity. Based on the level of xylanase production and crude enzyme activity features, one strain designated as *Bacillus* sp. detected as the best xylanase producer, and it was selected for the rest of the experiments. The optimization studies also were performed by altering the fermentation conditions and compositions of this basal medium under optimum shaking conditions.

### 2.5. Xylanase Assay

Oat-spelt xylan (95590) was used as the assay substrate for xylanase activity assessment. Enzyme activity was determined by measuring the release of reducing sugar during the enzyme-substrate reaction using 3, 5-dinitrosalicylic acid (DNS) stopping method [19, 20]. The reaction mixture for each enzyme assay contained 500 *μ*L of (0.5%) respective homogenized substrate prepared in phosphate buffer, pH 9.0, and 500 *μ*L appropriately diluted enzyme solution. Then the reaction was incubated at 55°C. After 20 min, 1 mL of DNS solution was added to the reaction mixture and boiled for 10 min. Followed by cooling in water for color stabilization. Absorbance of the solutions measured at OD_540_ nm by spectrophotometer (Model: JENWAY-Genova, Switzerland). The absorbance of reference samples (substrate solution incubated without enzyme and diluted enzyme solution in buffer) was deduced from the values of the test samples.

Xylanase activity was calculated by using D-xylose calibration curve. One unit of enzyme activity was defined as the amount of enzyme that catalyzes release of one micromole reducing sugar in one min from the prospective substrate under the standard assay conditions.

### 2.6. Identification of Microorganism

After sampling, isolation, and screening process, superior xylanase producer *Bacillus* sp strain was isolated from soil of cotton farm in Kashan-Iran. It was preliminary identified by morphological and biochemical tests [21, 22]. For sequencing analysis, the genomic DNA was extracted and purified from the strain by the standard chloroform isoamyl alcohol method using Roche kit [23, 24]. The amplification of the 16S rDNA was performed through PCR technique, using Taq DNA polymerase, genomic DNA as a template, and 3 forward and 5 reverse universal primers. The sequences of these primers used were as below:

3 F: 5′-AGAGTTTGATCCTGGC-3′
5 R: 5′-TACCTTGTTACGACTT-3′.

PCR products were delivered to SQ lab Co. (Germany). Receiving the sequencing results, 16S rDNA nucleotide sequence of the isolate has been deposited in GenBank, and aligned with the 16S rRNA sequences available in public databases in NCBI (National Center for Biotechnology Information, Available at: http://www.ncbi.nlm.nih.gov/), using BLAST (Basic Local Alignment Search Tool) software [7].

### 2.7. Parametric Optimization of Xylanase Production

(1) Carbon sources and the concentration of the carbon sources: various agricultural residuals such as wheat bran (WB), oat bran (OB), rice straw (RS), Bagasse, and Molasses were tested as sole substrate for xylanase production in fermentation medium, and the effect of different concentrations of the selected carbon source at 0.5%, 1%, and 2% (w/v) was studied on xylanase production. The enzyme production was carried out in flasks (250 mL), containing 50 mL of basic enzyme production medium supplemented with each agroindustrial byproduct (2% w/v) as substrates; after flask's sterilization, pH was adjusted to 8.0 using sterilized Na_2_CO_3_ (10%) and inoculated with 2% (v/v) 18 h grown superior* bacillus* inoculum and incubated at 37°C under shaking condition (180 rpm) in a shaker incubator. The 1 mL samples were removed at 18th h of fermentation and then in every 24 h intervals during 120 h SmF period, followed by enzyme extraction and xylanase assay procedure as previously described.

(2) Incubation period: SmF was carried out for 120 h fermentation process at 37°C, 180 rpm. The results find out the optimal time for maximum xylanase production in the presence of the best carbon source.

(3) Inoculum age and size: a 18-hour-old grown culture inocula in LB broth medium was used as inoculum at a level of 1, 2, 5, and 10% (v/v).

(4) Effect of nitrogen sources: production media devoid of any nitrogen sources were supplemented with different inorganic and organic nitrogen sources in the presence of optimum selected carbon sources, and the effect of various nitrogen sources was studied on xylanase production.

(5) pH: basic enzyme production medium of pH ranging from 6 to 11 was used for xylanase production. HCl (1 N) for pH 6.0 and sterilized Na_2_CO_3_ solution (10%) was utilized for pH adjustment ranging from 8.0 to 11.0.

(6) Incubation temperature: xylanase production was studied at temperatures of 32°, 37°, and 42°C during SmF.

(7) Agitation rate: SmF was carried out at agitation rates ranging from 160 to 220 rpm in a shaker incubator at 37°C, and xylanase production was studied.

(8) Effect of various additives: different additives like KH2PO4 (1 g/L), glycine (0.2% w/v), olive oil (0.2% v/v), SDS (0.2% v/v), tween 80 (1 g/L), D-xylose (1% w/v), glucose (1% w/v), fructose (1% w/v), and trace element solutions (pH 8) [5] were individually supplemented in the fermentation medium. Their effects on xylanase production were studied as enhancers or inhibitors.

### 2.8. Effect of pH on Activity and Stability of Xylanase

The influence of pH on xylanase activity was measured by incubating 500 *μ*L of appropriately diluted enzyme and 500 *μ*L of different buffers, adjusted to a pH of 7.0 to 10.6, containing oat-spelt xylan homogenized substrate (0.5% v/v). The buffers used were sodium phosphate (pH 7, 8, and 9) and glycine-NaOH (pH 10 and 10.6). The effect of pH on xylanase stability was measured over the pH ranging from 8.0 to 10.0 for 3 hr at 25° prior to the enzyme assay. After preincubating the crude enzyme without substrate at various pH values, residual xylanase activity at 30 min, 1, 2, and 3 hr time intervals was determined under optimal assay condition. 

### 2.9. Effect of Temperature on Activity and Stability of Xylanase

The effect of temperature on enzyme activity was studied by performing the standard assay procedure for 20 min at pH 9.0 (Optimum pH in assay condition) within a temperature range of 35°C to 65°C.

The thermal stability pattern of xylanase was determined by preincubation of crude enzyme without oat-spelt xylan substrate at temperatures ranging from 45° to 65°C for 30 min, 1, 2, 3, and 4 hr. After incubation, the enzyme extracts were cooled on ice for 10 min. Finally, the residual xylanase activity was measured at indicated time intervals for 4 hr under optimal assay condition.

### 2.10. Xylan Hydrolysis

Oat-spelt xylan hydrolysis analysis was evaluated after 48 h of fermentation (pH 8) at 37°C, by determination of reducing sugars and by high-pressure liquid chromatography. The samples were centrifuged at 10000 × g at 4°C for 20 min and filtered through a cellulose nitrate membrane filter (0.45 micrometer), to remove unhydrolysed xylan and other insoluble contaminants. Products of xylan hydrolysis were analyzed by HPLC, using an Aminex HPX-87H column (Biorad, 300 mm × 718 mm), at 40°C, with water Milli Q, as mobile phase (0.4 mL/min) and 20 *μ*L of sample injection to the column for a run time of 30 min. A LAChrom Refractive Index detector L-7490 was used [15]. 

The standard sugar was used (D-xylose, Merck) at 1 mg/mL as a reference.

## 3. Results

### 3.1. Isolation and Preliminary Screening of Xylanase-Producing Bacilli

40 strains were detected to produce xylanase, as displaying orange-colored xylan digestion halos on xylan agar plates with halo size equal to 18–35 mm in diameter.

### 3.2. Secondary Screening Results

Enzymatic hydrolysis of the surrounding xylan on the agar plates produced a clear zoon in the medium in a milky/cloudy background. Ten strains displaying biggest xylan digestion halo, equal in diameter to 25–35 mm, were screened.

### 3.3. Enzymatic Production

One *Bacillus *strain was selected as the best producer of extracellular xylanase, displaying xylan digestion halo diameter equal to 35 mm and maximum enzyme activity features at pH 9 and temperature 55°C.

### 3.4. Identification of Microorganism

The BLAST search of 16S rRNA gene sequence against sequences in nucleotide database has shown 100% homology with *B. moj *strain NBRC 15718 16S rRNA gene sequence with accession number of AB363735. Thus, the new indigenous isolated strain was identified and named as *Bacillus mojavensis* AG137. 

### 3.5. Optimization of Fermentation Medium for Xylanase Production

**Figure 1:**
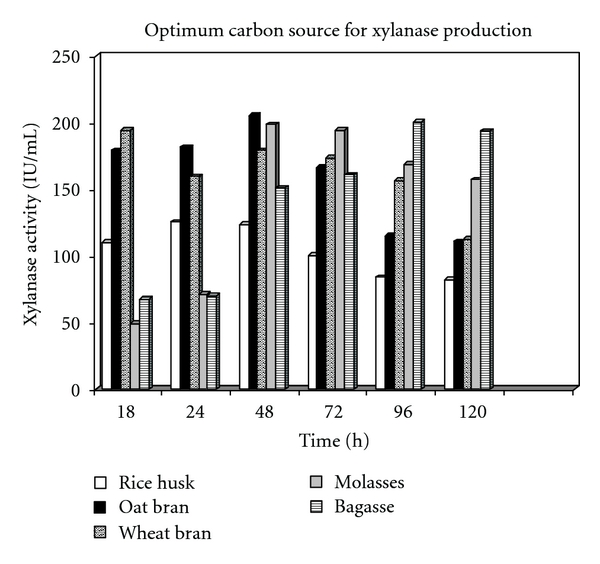
Effect of various carbon sources on xylanase production, accompanied by time course pattern for enzyme production.

Time course experiments indicated that the enzyme production reached its peak after 48 hr of SmF in the presence of oat bran as the best substrate for enzyme production, displaying the maximum enzyme activity equal to 205.415 IU/mL, closely followed by bagasse which results in enzyme activity equal to 200.321 IU/mL after 96 hr fermentation. The results are shown in Figure 1.

**Figure 2:**
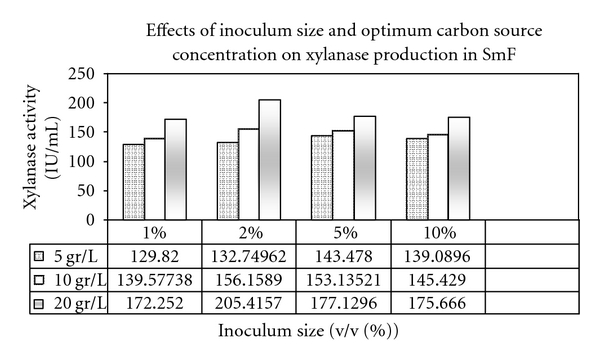
Effects of inoculum size and optimum carbon source concentration on Xylanase production in SmF.

In the presence of oat bran as optimum carbon source, xylanase production started approximately after 12 hr of fermentation, and the production peaked after 48 hr (205.415 IU/mL). After 96 hr of SmF, xylanase production still was acceptable (114.94 IU/mL), but declined thereafter to 110.803 IU/mL. The increase in xylanase activity during later stages in the medium might be due to the release of small amounts of xylanase from the aged cells, entering into autolysis. Also, it could be due to the scarcity of insoluble xylan particles in the medium which if present the culture broth might bind the xylanases [23]. Further incubation did not increase xylanase yield, but it resulted in a decline. Probably, the reduction in xylanase yield was due to the depletion of available nutrients to microorganism or due to the proteolysis [17]. The optimization step thus conducted in the presence of oat bran as the best enzyme inducer and optimum carbon source for xylanase production. Maximum xylanase activity was observed in the presence of oat bran when the production medium was supplemented with 20 g/L (2% w/v) oat bran as enzyme inducer substrate (Figure 2). What is more, xylanase production in SmF increased with an additional concentration of oat bran from 1% to 2% (w/v). Higher concentration of lignocellulosic substrates was not perfect according to the enzyme economical protocols [17]. Simultaneously, xylanase production was further increased to 205.415 IU/mL when 2% (v/v) of 18-hour-old inoculum of *B. moj* AG137 was inoculated to the production medium in the presence of 20 g/L (2% w/v) optimum carbon sources, but enzyme activity decreased to 175.66 IU/mL with further addition of inoculum size (Figure 2). Higher concentration of inoculums was not preferable for xylanase production in industrial fermentations [10]. Presumably, 5% inoculum level was so high that the nutrients were consumed faster, and it overall resulted in a lower enzyme yield (Figure 2). Hence, a balance between the proliferating biomass and available nutrients will yield maximum enzyme production [17].

Among all inorganic and organic nitrogen sources tested (Table 1), a combination of yeast extract and tryptone enhanced xylanase production to 249.308 IU/mL. Therefore, they were selected as optimum nitrogen sources for xylanase production by *B. moj *AG137. Likewise, the combination of yeast extract and beef extract in the production medium resulted in appropriate amount of xylanase activity equal to 213.218 IU/mL (Table 1). In contrast, ammonium sulphate alone and its combination with yeast extract resulted in strong repression of xylanase biosynthesis to a level of 74.714 IU/mL and 91.305 IU/mL (Table 1) as previously reported by Sa-Pereira et al. [15].

Enzyme production started in a fermentation medium adjusted to pH 6 and declined in media with pH adjusted to 9–11. Xylanase excretion displayed a sharp peak in enzyme activity equal to 265.401 IU/mL in the production medium adjusted to pH 8 (Table 2). 

The optimum aeration rate for xylanase production was achieved at 200 rpm showing enzyme activity equal to 290.76 IU/mL. Agitation rates below 200 rpm resulted in low xylanase yields. It is probably due to the difficulty in maintaining sufficient dissolved oxygen level for cell growth (Table 2). 

Optimum temperature for maximum xylanase production was found to be at 37°C, while displaying enzyme activity equal to 290.76 IU/mL (Table 2).

The influence of additives on xylanase production level has been reported in Table 3. Maximum increase in xylanase production equal to 302.46 IU/mL was observed in the presence of KH_2_PO_4_ followed by tween 80 equal to 265.45 IU/mL. Such compounds presumably increase the permeability of the cell membrane and cause rapid secretion of the xylanase. The effect of tween 80 to increase xylanase yield has been reported widely by Kapoor et al. [17]. In this study also the addition of Tween 80 to fermentation medium increased xylanase yield from *B. moj *AG137 strain. The possible reasons could be (i) increased enzyme stability and prevention of enzyme denaturation; (ii) affecting the substrate structure positively and making it more accessible for enzymatic hydrolysis; (iii) affecting enzyme-substrate interactions positively, leading to a more effective conversion of substrate. 

On the other hand, the ionic surfactants (SDS) cause cell membrane lysis [17], so it was used to see if cell lysis occurs in late growth hours and will release more xylanase from cell lysis or not? As expected, the addition of SDS to the fermentation medium resulted in a severe reduction of enzyme yield equal to 65.935 IU/mL (Table 3), and this might be caused due to multiple reasons such as conformational changes in the tertiary, secondary structure of the protein, binding of surfactants to the active site of the enzyme, or changing in the nature of the substrate by decreasing the availability of reaction sites [17].

The influence of olive oil on enzyme activity revealed that the olive oil alone did not enhance xylanase secretion via increasing cell membrane permeability significantly, but it inhibits xylanase production to a reduced level of 173, 228 IU/mL in combination with KH_2_PO_4_ during SmF; this fact could be due to the antagonistic interaction between these two compounds.

The recorded observations for xylanase activity in the production media, supplemented by sugars as additives (Table 3), indicate that D-xylose, sucrose, and glucose inhibited xylanase production strongly to minimum productivity equal to 11.31 IU/mL which could be caused by catabolism repression matter.

### 3.6. Effect of pH on Activity and Stability of Xylanase

**Figure 3:**
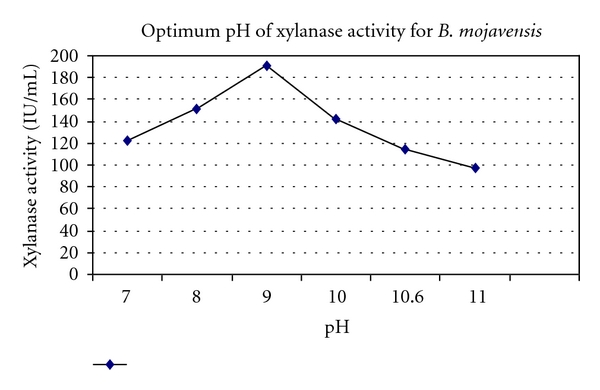
pH profile of xylanase produced from* B. mojavensis* AG 137.

**Figure 4:**
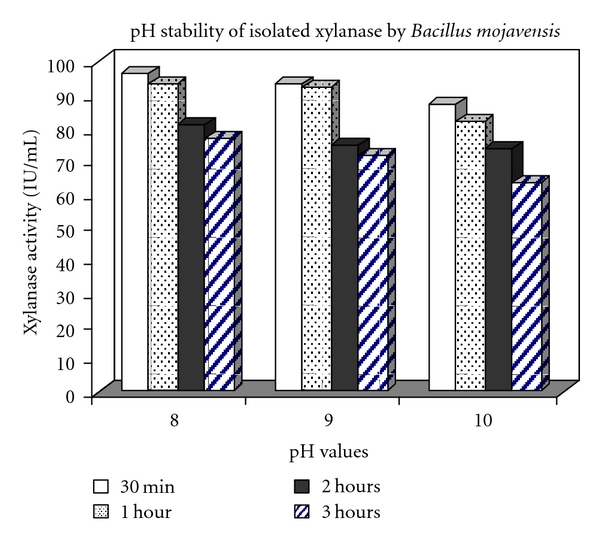
pH stability pattern of produced xylanase.

pH profile of xylanase activity is illustrated in Figure 3. Optimum pH was found to be 9 with activity level of 190.297 IU/mL. Xylanase pH stability pattern is illustrated in Figure 4. Attention to the results indicates that the enzyme preserves 75% and 74% of its initial maximum activity in pH 9 and 10 after 2 hours of preincubation, and it maintains 71% and 63% of its maximal activity after 3 hours of preincubation in pH 9 and 10.

### 3.7. Effect of Temperature on Activity and Stability of Xylanase

**Figure 5:**
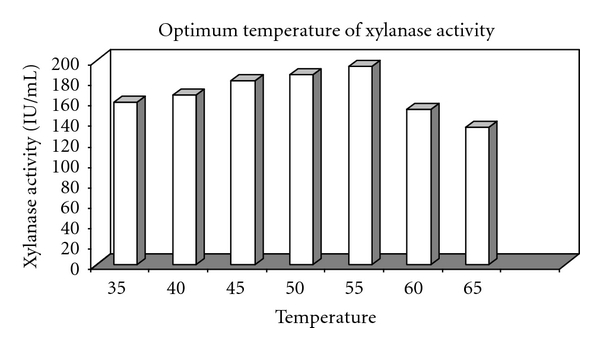
Temperature profile of produced xylanase from* B. mojavensis *AG 137.

**Figure 6:**
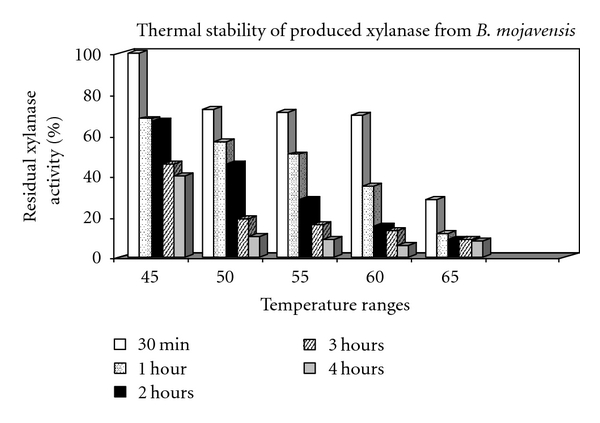
Thermal stability of xylanase produced by *B. mojavensis* AG 137.

Temperature profile of xylanase activity, showing a sharp peak of enzyme activity at 55°C equal to 194.68 IU/mL, is illustrated in Figure 5. Also, xylanase thermal stability pattern at optimum temperature of enzyme activity is illustrated in Figure 6. The preincubated enzyme preserves approximately 68, 56, and 50% of its initial activity at 45°, 50°, and 55°C for 1 hour. At 60°C, it presents 70% xylanase residual activity for 30 min. However, the most original xylanase activity was lost gradually at other elevated temperatures.

### 3.8. Xylan Hydrolysis Detection

**Figure 7:**
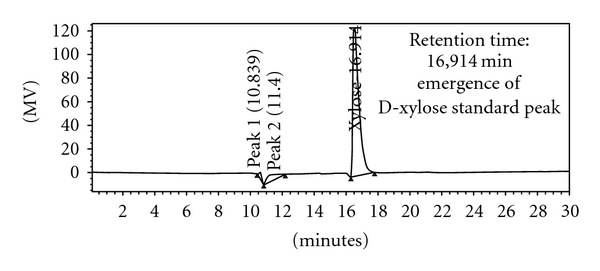
HPLC chromatograms of Standard D-xylose sugar sample.

**Figure 8:**
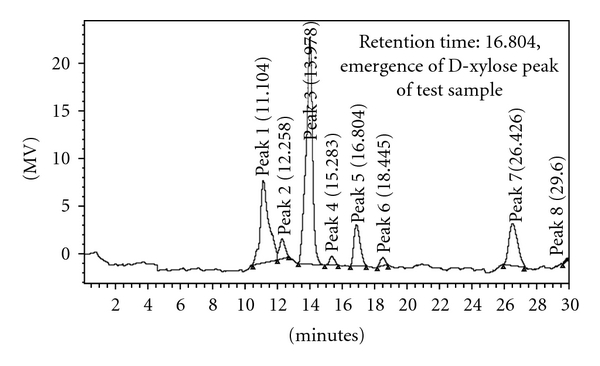
HPLC chromatograms of test sample.

Hydrolysis product of pure oat-spelt xylan substrate was confirmed through HPLC analysis. The results of HPLC chromatogram for standard D-xylose sugar in Figure 7 obviously display D-xylose peak in retention time of 16.91 min for the standard sugar. Likewise, the same D-xylose peak emergence in retention time of 16.8 min was observed in that of the test sample (Figure 8). Therefore, retention time equality of D-xylose peak in the HPLC chromatogram of the test sample to that of the standard sugar confirmed D-xylose sugar production as one of the released xylooligosaccharides during the standard assay condition in consequence of xylanase action on pure oat-spelt xylan substrate.

Hence, selective hydrolysis product of hemicellulase component of pure oat-spelt xylan substrate has been confirmed through HPLC analysis by the activity of this xylanase. Furthermore, the HPLC results validated that xylanase extracted from *B. moj* AG137 is a completely active, viable, and dynamic enzyme.

## 4. Discussion

Scientific records show that strains of* Bacillus mojavensis* have been implemented in biological control qualities. For instance, they are mostly known as very good antagonistic agents for the plant pathogen's fungus such as *Fusarium moniliforme* [25] Despite the fact that there are just few reports for *B. moj *strains, implemented in enzyme production area such as alkaline protease production from *B. moj* by Beg et al. [26], in this study, the production of alkaline xylanase by *B. moj* AG137 seems to be the first report. Regarding the fact that xylanase is categorized as an important industrial enzyme, it can be produced successfully by SmF [27] and SSF [1, 5, 13]. Although there are some reports on xylanase, high production equal to 500 IU/mL in the work by Poorna and Prema [28] and equal to 1300 IU/mL in the work by Kapoor et al. [17], among bacterial producers, 52 IU/mL of xylanase yield has been reported from *Bacillus v1-4* [16], or 17 IU/mL xylanase yield has been reported from *Bacillus thermoglucosidasius* [23]. In contrast, the presented results in this work and enhanced amounts of xylanase production demonstrate that among many methods to improve enzyme yield, optimization of medium components and cultivation conditions remains a facile and feasible one, supported by previous studies [17]. In addition, according to a study conducted by Oliveira et al. [14], among five agricultural wastes evaluated in SmF for xylanolytic enzyme production by *Penicillium janthinellum*, oat husk (oat bran) with xylanase production yield of 54.8 IU/mL was the best inducer of xylanase [14]. Similarly, the outcome of this study indicates that the optimization of SmF process parameters resulted in high level of xylanase production after 48 h of fermentation in the production medium supplemented with oat bran as the best enzyme inducer. Overall, a 1.55-fold increase in xylanase yield equal to 302.466 IU/ml has been achieved in comparison with xylanase activity equal to 194.68 IU/ml in nonoptimized fermentation medium. Moreover, the results highlight that oat bran supplemented optimized medium is very cost effective as compared to un-optimized oat-spelt xylan supplemented media. Alternative substrates such as bagasse and molasses also can be suitable substrate for xylanase production by this strain, so this finding accounts a positive point for this strain and its ability to ferment and produce sufficient amounts of xylanase in the presence of various lignocelluloses as it also has been supported by previous studies [17, 27]. Besides, high level of xylanase production on xylan-containing substrates such as oat bran, wheat bran, bagasse, and molasses suggests that xylan is necessary for the effective induction of xylanase from *B. moj *AG137.

Typically and according to previous studies, organic nitrogen sources have been found to stimulate xylanase production in *Bacillus* species [10]. 

To the best of our knowledge, using tryptone as one of the best organic sources in combination with yeast extract has not been reported earlier as optimum nitrogen sources for xylanase production so far, and this interesting new finding has been first reported in this study. Some main justifications for the superiority of selected nitrogen sources have been discussed further. It could be possible that the selected organic nitrogen provided by yeast extract and tryptone contains most of amino acids required for* B. moj *AG137 strain, and that these amino acids could be absorbed directly by the bacterial cells; what is more, complex nitrogen source like tryptone releases NH4 ions which stimulate growth. At the same time, it can increase enzyme yield because of its protease inhibiting nature at low concentration. On the other hand, ammonium sulphate alone and its combination with yeast extract resulted in strong repression of xylanase biosynthesis as previously reported by Sa-Pereira et al. [15].

 Thus, it can be concluded that xylanase excretion and its extracellular performance depend directly on type of ions present in solution since they affect overall kinetics, so (NH_4_)_2_SO_4_ causes an inhibition in xylanase excretion or synthesis in fermentation medium, in which (NH_4_)_2_SO_4_ ions probably inhibit enzyme production by altering and decreasing cell membrane permeability in *B. moj* AG137.

The pH of the medium strongly affects many enzymatic processes and transport of various components across the cell membrane [17]. Also, xylanase production by various bacteria and fungi has been reported to be markedly dependent on pH [10]. Acidic pH 4–6 generally favors fungal xylanases [12], while higher pH favors bacterial xylanases [28]. Agitation and aeration studies in this research were used to meet the demand of oxygen during fermentation process. Mechanical agitation, moreover, is reported to be crucial in SmF due to its effectiveness in mixing contents of media and prevention of cell clumping [17]. Thermal-alkali activity and stability of xylanases are very important properties due to their potential applications in several industrial processes. As case in point, paper and pulp industry is one of the distinguishing areas in which active and stable xylanase in high pH and temperature ranges is mighty pressing for their high quality of final products [10, 28, 29]. Accordingly, thermal and pH stabilities of xylanase, isolated from *B. caldoxylolgticus*, have been reported by Cordeiro et al. [23]. Lama et al. [3] also have reported a very high thermostable xylanase enzyme, isolated from *Bacillus thermantarcticus*. Furthermore, there are many other reports for extraction of alkali-tolerant xylanase from *Bacillus *sp. by Cordeiro et al. [23], thermostable xylanase from *Bacillus subtilis *by Sa-Pereira et al. [15], and stable xylanase report from *Bacillus pumilus* by Poorna and Prema [28]. 

In this study, considering enzyme pH and temperature activity and stability features (its high activity kept at pH 9 and 10 during 3 hours and being thermostable at temperatures ranging from 60–45°C for 3 hour) indicates that this xylanase from *B. moj* AG137 exhibits favorable potentials for application in industrial scale.

## Figures and Tables

**Table 1:** Effects of nitrogen sources on xylanase production.

Organic nitrogen sources	Xylanase activity (IU/mL)	Inorganic and combination of organic and inorganic nitrogen sources	Xylanase activity (IU/mL)
Peptone	118.118	NH_4_NO_3_	163.4742
Beef extract	157.1342	NaNO_3_	137.626
Casein	140.065	(NH_4_)_2_SO_4_	74.714
*Yeast extract	196.1496	Yeast extract and NH_4_NO_3_	193.223
Y + tryptone	249.308	Yeast extract and KNO_3_	185.4204
Y + beef extract	213.218	Yeast extract and NaNO_3_	180.89
Control (Y + peptone)	205.415	Yeast extract and (NH_4_)_2_SO_4_	91.305

*Y = *Yeast extract.

**Table 2:** Effects of microbial inoculum size, initial pH, agitation speed, and temperature of the fermentation medium on optimum xylanase production.

Optimum initial pH of fermentation medium	Xylanase activity (IU/mL)	Optimum agitation speed	Xylanase activity (IU/mL)	Optimum initial temperature of the medium	Xylanase activity (IU/mL)
6	215.657	160	189.329	32	186.39
7	148.332	180	252.721	37	290.76
8	252.721	200	290.76	42	229.31
9	182.981	220	220.534	—	—
10	49.842	—	—	—	—

11	32.285	—	—	—	—

**Table 3:** Effect of additives on xylanase production.

Additives	Xylanase activity (IU/mL)	Additives	Xylanase activity (IU/mL)
KH_2_PO_4_	302.466	Olive oil and KH_2_PO_4_	173.2281
Tween 80	265.4511	SDS	65.935
Olive oil	251.258	Glucose	14.7282
Trace elements solution	246.8696	Sucrose	11.802
Glycine	186.3957	D-xylose	11.314

Control (No additives)	252.722	—	—

## Abbreviations

SmF: Submerged fermentation
*B. moj*: *Bacillus mojavensis*.
